# Physiotherapy Protocol for Pain in the Immediate Postcesarean Postpartum: Randomized Clinical Trial

**DOI:** 10.1155/ogi/5179249

**Published:** 2026-06-15

**Authors:** Cristiane Rose Rossi Mazzoni, Maria Cristina Cortez Carneiro Meirelles, Ana Carolina Rodarti Pitangui de Araújo, Adriana Cristina Nicolussi, Mariana Torreglosa Ruiz

**Affiliations:** ^1^ Stricto Sensu Graduate Program in Health Care, Federal University of Triângulo Mineiro, Av. Getúlio Guaritá 107, Uberaba, Minas Gerais, 38025-440, Brazil, uftm.edu.br; ^2^ Department of Applied Physiotherapy, Federal University of Triângulo Mineiro, Av. Getúlio Guaritá 159, Uberaba, Minas Gerais, 38025-440, Brazil, uftm.edu.br; ^3^ Postgraduate Program in Rehabilitation and Functional Performance and Postgraduate Program in Nursing, University of Pernambuco, Av. Cardoso de Sá, s/n–Campus Universitário, Petrolina, Pernambuco, 56328-900, Brazil, ufpe.br

**Keywords:** cesarean section, pain, physiotherapy interventions, postoperative, postpartum period

## Abstract

**Objectives:**

To investigate the effectiveness of a physiotherapy protocol in preventing and managing pain in women in the immediate postpartum period after cesarean section.

**Methods:**

A randomized, parallel, open‐label clinical trial, whose data collection took place between October and December 2024, in the rooming‐in wards of a Brazilian teaching hospital. The study included 40 women in the immediate postpartum period after cesarean section, randomized into an intervention and a control group (standard care). Participants were asked about the presence of pain when performing movements. If pain was present, they were asked to identify its location, and a Visual Numerical Pain Scale was used to measure the pain score. Participants allocated to the intervention group received the physiotherapy intervention protocol, which consisted of postural guidelines. After 24 h, participants in both groups were asked again about the presence of pain in the listed activities. The chi‐square and Fisher’s exact tests were applied to categorical variables and the *t*‐test for independent samples for pain scores, and the effect size was calculated to determine the magnitude of the associations. A significance level of 5% was adopted for all analyses.

**Results:**

Pain complaints were reported in all movements assessed. Pain was felt most frequently in the abdominal region (generalized) and the surgical scar (localized). The intervention had an effect on the movements of coughing, sitting on the toilet, and walking. The physiotherapy protocol effectively reduced pain scores during walking (*p* = 0.009). Additionally, postpartum women in the intervention group reported greater satisfaction with pain management.

**Conclusion:**

The physiotherapy intervention protocol effectively reduced pain scores during walking, with effects on coughing and sitting on the toilet, and increased satisfaction with pain management.

**Trial Registration:** Brazilian Registry of Clinical Trials (ReBEC): UTN: U1111‐1308‐5716

## 1. Introduction

It is known that cesarean sections are a surgical procedure capable of saving maternal and infant lives when clinically indicated [1] and that studies have shown that rates above 10% do not lead to better results for maternal and fetal health [2, 3]. Therefore, given the potential complications inherent to the surgical procedure, the World Health Organization (WHO) recommends careful consideration and analysis of its indications [1].

Brazil stands out as one of the countries with the highest rates of cesarean sections. Between 2000 and 2019, the prevalence was 49%, compared to a global average of 21.1% [4]. In private healthcare, rates exceed 80%, while in public services, they range from 43% to 55% [5].

Furthermore, these rates are projected to increase worldwide, with estimates exceeding 30% of births by 2030 [1]. For this reason, the WHO has made efforts to publish new recommendations that justify the performance of the surgical procedure as an outcome of pregnancies [1].

It is estimated that more than 90% of women undergoing cesarean sections feel pain in the immediate postpartum period [6] and, despite the pharmacological analgesia used, incisional and abdominal pain, low back pain, neck pain, and discomfort in the lower limbs are frequent reports that hinder the woman’s rehabilitation and the performance of the activities necessary for self‐care and care of the newborn (NB) during this period [7, 8].

It is worth noting that pain is defined as “an unpleasant sensory and emotional experience associated with, or similar to, that associated with actual or potential tissue injury” [9], and its experience can directly impact activities of daily living and, consequently, quality of life. Regarding the postpartum period, the painful sensation can be even more impactful, given that the woman needs self‐care activities and care for the NB.

Pain at the surgical incision site is physiological. It occurs due to injury to abdominal wall tissue, which triggers an inflammatory response that releases various endogenous substances, such as histamine, one of the substances responsible for hyperalgesia and related to the biological adaptive process that facilitates tissue and scar repair. As a result of the injury and, consequently, the pain, there may be reduced bowel movements, gas accumulation, and restricted physical mobility, which impair postpartum recovery and interfere with proper positioning for breastfeeding, the care provided to the NB, and the self‐care of the postpartum woman [7]. Furthermore, these limitations can further worsen the symptoms and the painful sensation.

A study conducted with 30 postpartum women identified a negative correlation between cesarean section and breastfeeding, since all women presented inadequate posture, which can compromise breastfeeding due to the cycle of pain and discomfort caused by these postural changes [10]. Trunk flexion is a common inadequate postural habit adopted during breastfeeding and, when combined with shoulder asymmetry, can cause discomfort and generate muscle tension [11], thereby compromising the breastfeeding experience.

Pain in the abdominal scar resulting from surgery can hinder lymphatic drainage and limit movement due to the presence of pain and/or fear of feeling pain, encouraging longer periods of bed rest and favoring the appearance of lower limb edema. Furthermore, approximately 50% of women report some type of pain during the postpartum period. During this time, when women are overwhelmed with daily activities, care must be taken with body movement, avoiding postures that protect against pain, so that the postpartum woman does not experience musculoskeletal discomfort, which will negatively influence her well‐being [7].

In this scenario, physiotherapy can aid in managing pain complaints using various resources [7]. Although descriptions of several nonpharmacological resources used by physiotherapists exist, evidence of physiotherapeutic interventions applied in the immediate postpartum period, through the stimulation of movement aimed at preventing postures that cause pain, is scarce.

Given the high prevalence of cesarean sections and associated pain complaints and the scarcity of studies and protocols for physiotherapeutic interventions for pain relief, this study is justified.

The study tested the alternative hypothesis: The application of a physiotherapeutic intervention protocol aimed at stimulating early movement helps to reduce pain scores reported by postpartum women who had a cesarean delivery compared to pain scores reported by postpartum women who did not receive the intervention.

The objective of this study was to evaluate the effectiveness of physiotherapeutic interventions in preventing and managing pain in women in the immediate postpartum period after a cesarean section.

## 2. Material and Methods

### 2.1. Study Design

This is a randomized, parallel, and open clinical trial, entitled “Stimulating movement and Physiotherapy interventions to prevent post‐cesarean pain”. The clinical trial protocol was registered in the Brazilian registry of clinical trials. The recommendations of the Consolidated Standards of Reporting Trials [12] were strictly adopted in the study.

The choice of an open‐label design stemmed primarily from limitations inherent to the intervention being evaluated. Because it was a physiotherapy intervention with perceptible characteristics, blinding the participants and the applying professional was not feasible, since both necessarily recognize the intervention received or administered.

Regarding blinding the outcome assessor (single‐blind), although methodologically desirable to reduce detection bias, its implementation proved operationally limited in this study. The same physiotherapist responsible for the intervention also collected the data due to logistical constraints and the need to standardize the application of assessment instruments, especially those that require specific training. Introducing an independent assessor would imply increased inter‐rater variability and a potential compromise of measurement consistency.

Nevertheless, strategies were adopted to mitigate the effect of expectation, both on the part of the participants and the researcher: (1) use of validated and standardized instruments for pain measurement, reducing interpretive subjectivity; (2) application of rigorous and predefined protocols for data collection, minimizing evaluator interference; and (3) neutral guidance to participants, avoiding the induction of expectations regarding the superiority of any intervention, and performance of statistical analyses based on repeated measures, which contributes to reducing the impact of individual variations.

### 2.2. Inclusion and Exclusion Criteria

All postpartum women who had undergone cesarean section, were over 15 years of age, were hemodynamically stable, conscious, oriented, in good clinical condition as described in the medical records, after having the urinary catheter removed, who had eaten after surgery, and whose procedure had been performed more than 8 hours and no more than 24 h previously were included in the study. The inclusion limit of no more than 24 h was justified to ensure sufficient time for performing the intervention and follow‐up before hospital discharge, typically within 48 h in physiological cases.

Women who presented complications/intercurrences, clinical contraindication, or medical diagnosis of cognitive impairment that prevented participation, as well as those who required the use of opioid medications before allocation, were not included.

Women undergoing the intervention who required opioid use in the interval between allocation and follow‐up (24 h later) were considered losses.

No participants were excluded after randomization in this study, and no losses were recorded.

### 2.3. Setting

The study was conducted in the rooming‐in units of a maternity hospital in the interior of Minas Gerais. It is a public teaching hospital linked to a federal university, a reference for resolving high‐risk pregnancies and infectious diseases in the pregnancy–puerperal cycle. The rooming‐in unit has 12 beds, and according to institutional statistics from 2024, 1310 births were recorded this year, and 714 (54.5%) were cesarean sections.

### 2.4. Trial Procedures

#### 2.4.1. Procedures Prior to Data Collection: Validation of the Intervention Protocol, Data Collection, Educational Materials, and Pilot Study

Initially, a scoping review was conducted on the use of physiotherapeutic interventions to reduce pain after cesarean section, with searches conducted in November 2023. This review highlighted a range of physiotherapeutic resources and provided positive evidence of the interventions. However, a scarcity of studies on the subject was observed.

Through reading the articles during the review, a key article was identified. Through manual searching, it was possible to retrieve six additional publications that identified relevant interventions for this study’s composition of the intervention protocol.

The protocol of physiotherapeutic interventions for pain relief in women in the immediate postpartum period after cesarean section initially consisted of 12 items and was submitted to validation by experts.

The experts were selected in May 2024 based on an analysis of the information contained in their Lattes Curriculum. This group included researchers in the field of physiotherapy who had training and experience in women’s health. The invitation was made by email, and the experts were also invited to suggest contacts who worked on the topic (snowball technique). In total, 39 experts were selected and invited. The final sample consisted of 13 experts, following the recommendations in the literature, which recommends 6–20 validators and a minimum of three individuals when representing a professional group [13].

The inclusion of experts followed the criteria of Guimarães et al. [14], based on the expert’s clinical experience. As a criterion for inclusion in the study, the expert should obtain at least four points, that is, have clinical experience in the subject area, as suggested by the classification’s authors [14].

Experts who did not respond within 15 days of receiving the instrument were not included. Exclusion criteria were incomplete responses to the items and a Guimarães classification score of less than four points. One participant identified through the snowball sampling technique was excluded from the analysis because they had only 2 years of professional experience.

The validation questionnaire, accompanied by a statement clarifying the study’s objectives and a descriptive document outlining the activities requested of the experts, was sent online via a Google Forms form.

The validation instrument consisted of two parts. Part I involved characterization data on the experts, such as age, gender, academic degree, education, length of time since graduation, whether they were teaching, and, if so, for how long. Part II described the validation items of the intervention protocol. Each of the validation items was evaluated based on a Likert scale containing the options “*strongly disagree*,” “*partially disagree*,” “*disagree*,” “*agree*,” “*partially agree*,” and “*strongly agree*.” At the end of the instrument, the experts had a blank field for free‐form entries.

The collected data were imported from Google Forms into a Microsoft Excel database. They were then imported into the Statistical Package for the Social Sciences (SPSS) program, Version 23.0, for processing and analysis. The agreement between experts was analyzed using the Content Validity Index (CVI) [15].

The responses “strongly agree,” “partially agree,” and “agree” were grouped as agreement, and “strongly disagree,” “partially disagree,” and “disagree” were grouped as disagreement. The CVI was calculated using the following formula: CVI = agreement/total number of experts. A minimum coefficient of 0.80 was adopted as the relevance index for the agreement among validators [15].

Most of the experts held doctoral degrees, specialized in women’s health physiotherapy, and worked as professors. Applying Guimarães’ criteria, all were eligible for the study.

The protocol was validated in its first version. Items with a CVI lower than 0.80 were removed from the instrument, and those with a CVI higher than 0.80 were maintained. It was decided not to make adjustments but to remove items that could generate doubts. Thus, a new round of validation was not necessary. The final version of the instrument consisted of ten items: (1) breastfeeding lying down; (2) changing from lying down to standing next to the bed; (3) walking, (4) changing and bathing the NB; (5) breastfeeding/feeding in the sitting position; (6) sitting/standing up from the chair; (7) sitting/standing up from the toilet; (8) coughing; (9) bathing, and (10) active movement of the feet.

At the same time, a folder was prepared and validated for distribution to all participants. It consisted of illustrated guidelines to adopt postural changes after hospital discharge to reduce postoperative pain. Eight experts who also participated in the previous stage were involved in the validation, and the same procedures were followed. All items obtained a CVI greater than 0.80, so a new validation round was unnecessary.

After validation of the intervention protocol, data collection instruments for the control group (CG) and the intervention group (IG), and educational materials, a pilot study was conducted with 20 women, 10 allocated to the IG and 10 to the CG. The change from lying down to standing position was the one that obtained the highest frequency of reports, and the result was chosen due to its magnitude. In the IG, the mean score obtained was 5.55 ± 3.67, and after the intervention, it was 2.11 ± 2.52; in the CG, the mean score at allocation was 6.50 ± 3.27, and 24 h later, a mean score of 5.40 ± 3.09 was reported. The data were collected during the month of September 2024. The pilot study allowed the feasibility of the study to be tested, and the sample calculation for the clinical trial was performed.

### 2.5. Data Collection

Every day, the researcher went to the wards and asked the nurse in charge for the daily census of patients assisted in the unit. From the list, the researcher verified eligibility and noninclusion criteria in the medical records.

The data collection instrument was standardized to be applied at least 8 h after the cesarean section, the removal of the indwelling urinary catheter, and the first feeding. These criteria aimed to respect the initial postpartum rest, overcome the difficulty of performing movements while using the catheter, and avoid the consequences of a long period without eating. The limitation of the first contact to 24 h occurred to ensure the follow‐up necessary for the study.

Eligible postpartum women were informed about the objectives of the study, and those who agreed to participate signed the informed consent form (ICF) or the free and informed assent (FIA) form. It is important to note that the researcher emphasized the possibility of being assigned to the control or IG, depending on randomization, before signing the form.

The data were collected between October and December 2024 by the principal investigator using standardized interviews, and all participants received identical instructions from the researcher.

### 2.6. Description of the Intervention

The study intervention applied the physiotherapeutic intervention protocol for preventing and managing postcesarean pain, constructed from the data obtained in the scoping review and methodological study described above. The postpartum women allocated to the IG were asked to perform the following activities described in the protocol: (1) breastfeeding while lying down; (2) changing from a lying position to standing next to the bed; (3) walking; (4) changing and bathing the NB; (5) breastfeeding/feeding in a sitting position; (6) sitting/standing up from a chair; (7) sitting/standing up from the toilet; (8) coughing; (9) bathing; and (10) active foot movement. Item 10 was not assessed in isolation, since foot movement is present in most of the other required activities. It was not necessary to follow the sequence of movements but to perform all of those listed.

During the movement, the postpartum woman was asked about pain complaints. When the woman reported pain, the Visual Numerical Pain Scale (VNS) was used to assess pain, ranging from 0 (*no pain*) to 10 (*worst possible pain*) [16]. With the aid of a body image illustration, the postpartum woman was asked to indicate the painful location during movement.

After performing the movements and indicating the presence or absence of pain, the postpartum woman then received instructions from the researcher on the need for postural changes to perform each activity. The guidance outlined the most appropriate way to perform the movements, including the recommended rest time between movements, and advised against overloading the abdominal region and surgical scar to minimize pain.

The instructions included verbal explanations, a demonstration of the postural changes by the researcher, followed by the postpartum woman herself performing the movement. The intervention lasted an average of 30 min, while data collection in this group lasted 50 min. When only data were collected, the average duration was 15 min. The intervention protocol is available in the Supporting Information (available here).

After applying the interventions, the woman’s satisfaction was assessed using a Likert scale: How did you feel about the guidance on performing the activities? The postpartum woman could choose the following answers: (1) not satisfied; (2) somewhat satisfied; (3) satisfied; and (4) very satisfied.

After the intervention, the researcher emphasized that she would return the following day (24 h after the intervention) for follow‐up and completion of the study.

After 24 h from the first assessment, the researcher returned to ask if the woman could still perform the movements and to record the pain scores for each of the movements described. If the postpartum woman reported pain, she was asked to indicate the location and the pain score.

At the end of the collection, the researcher thanked her for participating and handed her the folder containing postural guidelines for improving pain complaints, which will be used after hospital discharge. It is worth noting that the postpartum women in the IG received standard institutional care throughout their hospitalization period.

At the institution, patients do not receive structured instructions either before or after surgery. There are no physiotherapists dedicated exclusively to assisting in the sector, and activities with undergraduate physiotherapy students are conducted sporadically in the unit. Thus, standard care for postpartum women assisted in the unit consists of assessing pain complaints and, when present, administering pharmacological analgesia, most frequently using nonopioid analgesics and nonsteroidal anti‐inflammatory drugs. If more potent analgesics are needed, a medical evaluation is requested. There is no prescribed analgesia schedule; it is administered based on the patient’s complaint.

For the postpartum women allocated to the CG, data collection occurred the same way as in the IG, except for the application of the intervention. After allocation, the researcher thanked the postpartum woman for participating and informed her that she would return in 24 h for the reassessment. Pain complaints, location, and scores were reassessed during this period. As in the IG, the folder was delivered 24 h after recruitment. The postpartum women in the CG received standard institutional care throughout the hospitalization period.

The principal researcher administered the intervention and conducted the assessment at various times.

### 2.7. Trial Outcomes

The primary outcome was the intensity of postoperative pain, assessed 24 h after allocation to the CG or IG. Pain was measured using a VNS from 0 to 10 during the execution of nine standardized movements defined in the study protocol. For analysis, the pain scores obtained in the nine evaluated movements were considered. The independent variable consisted of applying the physiotherapeutic intervention protocol for the prevention and management of postcesarean pain.

### 2.8. Sample Size Calculation

The pain score before and after 24 h of allocation was considered for the sample calculation. In the IG, the mean obtained was 5.55 (±3.67), and after the intervention, it was 2.11 (±2.52). In the CG, the mean score at allocation was 6.50 (±3.27) and, after 24 h, it was 5.40 (±3.09). A significance level of 5% (*α* = 0.05), a statistical power of 90% (1 − *β* = 0.90), and an estimated effect size of 1.17, calculated based on the standardized difference between the groups (Cohen’s d), were considered. The calculation was performed using the G∗Power software (Version 3.1.9.7), through the “Means: Difference between two independent means (two groups)” test, with a priori analysis for bilateral testing. The result indicated the need for 19 participants per group, totaling 38 participants, to ensure adequate statistical power to detect the observed difference. Based on the sample calculation, 40 postpartum women were allocated, 20 in the IG and 20 in the CG.

### 2.9. Randomization

After obtaining the consent of the postpartum women, the researcher contacted the designated randomization center for the study, which was responsible for managing the allocation groups. This center had the randomization lists prepared for the study in the SPSS software, Version 23.0.

The researcher communicated with the center’s members through the WhatsApp application to verify the order in which the postpartum women were included in the study and their respective allocation group (IG/GC). The simple randomization technique was employed, and it is worth noting that the center’s members were not part of the study team.

### 2.10. Statistical Analysis

The data were entered into a Google Forms form, imported into an Excel spreadsheet, and then imported into the SPSS software, Version 23.0.

A descriptive analysis of the data related to sociodemographic, clinical, and obstetric variables (absolute numbers and percentages, mean, standard deviation, and minimum and maximum values) was performed. The chi‐square and Fisher’s exact tests were used to test the hypothesis of homogeneity of the two groups (IG and CG). The *t*‐test for independent samples was used to compare the differences in scores between the groups. The chi‐square and Fisher’s exact tests compared satisfaction with postoperative pain management and control. The calculations of prevalence ratios and respective confidence intervals were 95%. A significance level of 5% was adopted for all analyses.

The effect size was calculated to determine the magnitude of the associations. Cohen’s *d* test was applied for groups with homogeneous variance, Hedges’d was used for samples with fewer than 20 subjects, and Glass’s delta was used when there was heterogeneous variance between groups. For the interpretation of effect size, Sawilowsky’s classification (2009) was used [17].

### 2.11. Ethical Aspects

The study obtained ethical approval from the Ethics Committee, under opinion no. 6,843,708 on May 23, 2024. The study was guided by the Guidelines and Regulatory Norms for Research involving human beings, contained in National Resolution 466/2012/CNS/MS, as well as the ethical principles contained in the Declaration of Helsinki, ensuring that all participants of the study signed the free and ICF.

## 3. Results

**FIGURE 1:**
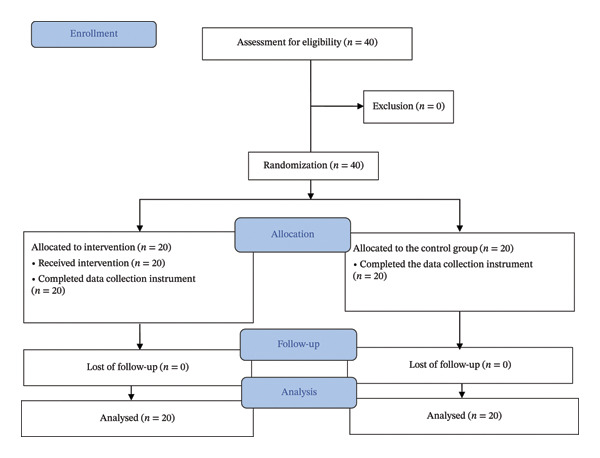
Flowchart CONSORT 2010.

Forty postpartum women were equally distributed between the IG and CG. There were no exclusions or losses. The study flowchart is shown in Figure 1.

Table 1 presents the baseline data of postpartum women at the time of allocation, according to study groups. From the base data, it is identified that there are no significant differences between the groups, as shown in Table 1.

**TABLE 1:** Comparison of baseline information of postpartum women in the two groups.

Variable	Total *n* = 40 (%)	Intervention group *n* = 20 (%)	Control group *n* = 20 (%)	*p*
Maternal age	28.80 ± 6.91	27.60 ± 7.10	30.10 ± 7.00	0.552
Self‐declared black or brown skin color	33 (82.5)	13 (68.4)	20 (100.0)	0.091
Lives with a partner	35 (87.5)	16 (80.0)	19 (95.0)	0.342
Education level higher than complete secondary education	23 (57.5)	11 (55.0)	12 (60.0)	1.000
Income greater than $500.00	27 (67.5)	13 (65.0)	14 (70.0)	1.000
Paid occupation	17 (42.5)	09 (45.0)	08 (40.0)	1.000
Smoking	10 (25.0)	06 (30.0)	04 (20.0)	0.716
Alcoholism	04 (10.0)	03 (15.0)	01 (5.0)	0.605
Comorbidities	15 (37.5)	09 (45.0)	06 (30.0)	0.514
Adequate prenatal care	36 (90.0)	18 (90.0)	18 (90.0)	1.000
Primiparous	09 (22.5)	05 (25.0)	04 (20.0)	1.000
Previous cesarean section	14 (35.0)	06 (30.0)	08 (40.0)	0.741
Delivery complications	04 (10.0)	03 (15.0)	01 (5.0)	0.605

All participants received nonsteroidal anti‐inflammatory drugs (ketoprofen) and a nonopioid analgesic (dipyrone), with no difference in consumption between the groups. However, none of the participants required the use of opioid analgesics, which were prescribed only when necessary.

There was an increase or no change in scores over time among postpartum women with postoperative pain complaints, except for reductions in movements associated with breastfeeding while lying down (2.00 points) and coughing (0.85 points) in the CG. In the IG, there was a 3.33‐point reduction in scores for the walking movement after 24 h.

A statistically significant difference was observed in pain during the coughing movement (*p* = 0.027) in the CG and during walking in the IG (*p* = 0.009). Therefore, the intervention effectively reduced pain scores during postoperative walking, as shown in Table 2.

**TABLE 2:** Comparison of pain scores in the intervention group (IG; *n* = 20) and the control group (CG; *n* = 20) before and after the intervention, Uberaba, MG, Brazil, 2024.

Movement	Group	*n*	Mean	SD^∗^	CI 95%^†^	*p* ^∗∗^	Effect size	Interpretation^#^
Breastfeeding while lying down	IG**^‡^**	02	3.50	0.707	−16.50–5.50	0.099	7.78	Very large
	CG^§^	01	−2.00					

Changing from lying down to standing next to the bed	IG	18	1.69	2.213	−2.86–1.63	0.174	0.54	Medium
	CG	18	0.57	1.949				

Breastfeeding/feeding in a sitting position	IG	01	< 0.001		−3.25–2.75	0.444	−0.41	Small
	CG	03	1.66	1.527				

Sitting/standing up from the chair	IG	10	1.30	2.983	−3.05–1.69	0.575	0.23	Small
	CG	13	0.69	2.136				

Coughing	IG	08	1.62	1.302	−5.39–2.04	**0.027**	1.31	Very large
	CG	07	−0.85	2.193				

Changing and bathing the NB	IG	—			—	—	—	—
	CG	01	6.00					

Sitting/standing up from the toilet	IG	05	3.00	2.236	−3.62–3.59	0.151	1.05	Large
	CG	03	0.66	1.154				

Bathing	IG	01	< 0.001		—	—	—	—
	CG	—						

Walking	IG	03	−3.33	1.527	1.42–6.44	**0.009**	−2.43	Very large
	CG	05	0.60	1.342				

*Note:* Bold indicates *p* values < 0.05.

^∗^SD = standard deviation.

^‡^IG = intervention group.

^§^CG = control group.

^†^CI 95% = confidence interval 95%.

^∗∗^ *p* = *p* value.

^#^based on Sawilowsky’s classification (2009).

When examining the magnitude of the differences, based on the effect size, it is evident that the intervention showed a very large effect on coughing and walking movements and a large effect on the sitting‐on‐the‐toilet movement, as shown in Table 2.

Pain complaints were reported in all listed movements, both at the time of allocation and 24 h later. The movement required to change from the lying down to standing position (*n* = 36, 90.0%) was the one with the highest number of complaints reported both at the time of allocation and 24 h later (*n* = 29, 72.5%), followed by the movement of sitting down and getting up from the chair (allocation: *n* = 30; 75.0%; 24 h later: *n* = 26; 65.0%).

The movement required to perform care for the NB, such as changing diapers or performing an immersion bath, presented a lower frequency of pain reports and remained constant at both times (allocation: *n* = 03; 7.5%; 24 h later: *n* = 03; 7.5%); however, an increase in the mean (5.67 ± 4.16) was observed throughout the evaluation time (7.00 ± 1.00).

The coughing movement was more intense during the allocation (8.42 ± 1.46) and 24 h after surgery (8.18 ± 1.68) and was reported as severe pain.

There was a reduction in the frequency of pain complaints during movements 24 h after the allocation, except for the movements of coughing (allocation: *n* = 19; 47.5%; 24 h after: *n* = 22; 55.0%) and sitting down and getting up from the toilet (allocation: *n* = 17; 42.5%; 24 h after: *n* = 18; 45.0%).

A detailed description of the pain reported during movement is provided in Table 3.

**TABLE 3:** Distribution of absolute and relative frequencies regarding pain when performing functional activities before and after 24 h, considering the intervention group (IG; *n* = 20) and the control group (CG; *n* = 20), Uberaba, MG, Brazil, 2024.

Variable	Intervention group		Control group	
	Before	24 h later	Before	24 h later
	*n* (%) Mean ± SD	*n* (%) Mean ± SD	*n* (%) Mean ± SD	*n* (%) Mean ± SD
Pain when breastfeeding while lying down	07 (35.0) 6.57 ± 2.07	03 (15.0) 6.67 ± 2.88	07 (35.0) 8.14 ± 1.57	02 (10.0) 6.00 ± 2.82
Pain when changing from lying to the standing position	18 (90.0) 6.83 ± 2.03	14 (70.0) 5.64 ± 1.78	18 (90.0) 7.33 ± 2.02	15 (75.0) 6.53 ± 1.51
Pain when breastfeeding in a sitting position	07 (35.0) 6.57 ± 1.71	01 (5.0) 10.00	09 (45.0) 6.67 ± 1.73	08 (40.0) 6.00 ± 2.20
Pain when changing from a sitting to a standing position	14 (70.0) 6.57 ± 2.02	12 (60.0) 5.58 ± 2.39	16 (80.0) 6.56 ± 1.93	14 (70.0) 6.00 ± 2.08
Pain when coughing	10 (50.0) 8.50 ± 1.50	10 (50.0) 7.20 ± 1.81	09 (45.0) 8.33 ± 1.50	12 (60.0) 9.00 ± 1.04
Pain when caring for the NB	02 (10.0) 8.00 ± 1.41	02 (10.0) 7.00 ± 1.41	01 (5.00) 1.00	01 (5.0) 7.00
Pain when sitting and getting up from the toilet	10 (50.0) 6.40 ± 1.95	08 (40.0) 5.38 ± 2.72	07 (35.0) 7.29 ± 1.79	10 (50.0) 6.30 ± 1.83
Pain when taking a shower	11 (55.0) 7.64 ± 2.24	04 (20.0) 5.75 ± 2.63	07 (35.0) 5.86 ± 2.47	01 (5.0) 8.00
Pain when walking	11 (55.0) 6.36 ± 2.16	05 (25.0) 6.00 ± 2.45	08 (40.0) 7.38 ± 2.07	07 (35.0) 5.86 ± 2.27

Abbreviation: SD = standard deviation.

In all movements evaluated, the pain was characterized as moderate to severe, most frequently located in the abdominal region (generalized) and at the surgical scar site. Only breastfeeding while lying down or sitting contributed to pain in the thoracic and cervical areas.

When comparing satisfaction with pain control for both groups, both groups reported satisfaction or very satisfied. However, it was found that, in the IG, there was a statistically significant association (*p* = 0.031) between receiving guidance and feeling very satisfied. Participants who received the intervention were 3.41 times more likely to be very satisfied with the care and pain control, as shown in Table 4.

**TABLE 4:** Association between level of satisfaction with pain control between the intervention and control groups, Uberaba, MG, Brazil, 2024.

Satisfaction	IG *n* (%)	CG *n* (%)	PR	CI 95%	*p*
*Very satisfied*	18 (90.0)	11 (55.0)	3.41	(0.94–12.35)	0.031
*Satisfied*	02 (10.0)	09 (45.0)	0.46	(0.27–0.46)	

## 4. Discussion

Pain complaints were reported in all listed movements, both at the time of placement and 24 h later, of moderate or strong intensity, and predominantly in the surgical scar and abdominal region. Postcesarean recovery shares several similarities with recovery from nonobstetric abdominal surgery, especially regarding the response to inflammatory stress resulting from tissue damage, physiological reactions caused by blood loss, and changes in fluids, but there is also pain after delivery [18].

In obstetrics, pain is one of the factors that impact the quality of functional recovery, interfering with self‐care, NB care, and breastfeeding [19]. A study carried out to measure the intensity of pain in women in the immediate postpartum period following a cesarean section resulted in the majority classifying the pain as moderate, and less than 5% of participants classifying the pain in the surgical wound as intense [10]. Another study conducted in Denmark found that postpartum women described the first day after a cesarean section as extremely painful, with a desire for an early rehabilitation plan [20]. This painful experience is associated with the surgical inflammatory process, abdominal distension, and activation of local nociceptors, which justifies the need for therapeutic strategies that mitigate these effects [21, 22].

In the present study, the transition from lying down to standing caused a significant increase in pain, especially in the first few days. This can be justified by the involvement of the abdominal muscles and pelvic floor in stabilizing the trunk during vertical movement, in addition to the sensitivity of the surgical scar and muscle stiffness induced by postoperative immobility [21, 22].

In this sense, a systematic review study concluded that physiotherapeutic resources such as electrotherapy, massage therapy, and postural guidance were effective in treating complications after a cesarean section, with special emphasis on reducing pain during activities of daily living, comfort level, joint range of motion, and reduction in analgesic consumption [23], indicating the relevance of this study that aims to apply a protocol of therapeutic interventions for the prevention and management of postpartum pain.

In this study, although the participants reported pain in all movements, the transition from lying down to the standing position significantly increased pain complaints in both groups, both before and 24 h after the intervention. A similar result was described in a study carried out in Denmark with postpartum women who underwent a cesarean section, in which the movement from lying down to the sitting position and getting out of bed were identified as particularly painful [20]. This happens because work is required from several muscle groups when getting up from a lying position. The main muscles are the leg muscles, which propel the body upwards, and the core muscles (abdomen and back), which stabilize the trunk and ensure an upright posture. In addition, the gluteal muscles come into play to assist in hip extension. As previously mentioned, the surgical inflammatory process can compromise the execution of the movement and cause a painful sensation [21, 22].

In the present study, the activity of breastfeeding while lying down or sitting generated reports of pain in the thoracic and cervical regions, unlike those commonly reported in other activities. Women who have undergone a cesarean section tend to have greater compensation for breastfeeding, since the presence of the surgical wound limits changes in position, and the adoption of appropriate postures for breastfeeding can favor the appearance of cervical pain and lumbago [7]. This may be related to the sustained flexion of the head and trunk and the lack of adequate support, commonly adopted by postpartum women to monitor the NB during long periods of breastfeeding, in addition to the increase in breast weight in the postpartum period, which contributes to muscle overload and chest pain [11]. It is noteworthy that the posture, when inadequate, becomes uncomfortable and can generate muscle tension, contributing to cervical and chest pain and consequently interfering with lactation [24].

A reduction in pain scores during this movement was observed in the CG, with a very large effect size but without statistical significance. Thus, it is suggested that when evaluating pain scores during breastfeeding in a seated position, studies with larger sample sizes should be conducted.

Caring for a NB was the activity that presented the lowest frequency of reported pain and remained constant even with an increase in the average over time. A study conducted in China with 432 women between six and eight weeks postpartum identified wrist pain as the second most prevalent discomfort, attributed to the physical effort associated with caring for the child, such as holding, lifting, and breastfeeding, concluding that these activities increase the likelihood of experiencing postpartum pain [25]. When comparing the results, it should be considered that in the present study, the assessments were carried out in the immediate postpartum period, and in the Chinese study, they were carried out in the late postpartum period. However, we can infer that pain may be present over time and especially throughout the postpartum period, thus reinforcing the need for research on nonpharmacological alternatives for pain relief for women.

Sitting down and getting up from a chair was the second most frequently complained of pain and had similar results to the movement of sitting down and getting up from the toilet. Walking had a lower frequency of pain reports at placement and was reduced substantially after 24 h. This can be explained by the stimulation of early mobility, which improves local circulation and reduces sensitivity to pain [26].

In all movements, the site of pain in most reports was the surgical scar. Studies indicate that 100% of postcesarean women report mobility limitations due to pain when sitting and standing, while 75% report pain when walking [26], similar to the results obtained in the study.

Higher pain scores were observed when coughing 24 h after delivery. Coughing is one of the main forms of pulmonary defense and aims to keep the airways clear, and after surgeries, it is common for it to be inhibited by the patient, either out of fear or due to the pain itself [27]. Strategies such as containing the scar with pillows while coughing should be taught as part of the orientation protocol, substantially reducing pain scores [27]. Therefore, it is necessary to train women to adapt to the experience of coughing.

It is noteworthy that the guidelines from the applied protocol were effective in reducing cough pain scores and should be implemented in clinical practice.

In this study, physiotherapeutic interventions effectively reduced pain scores 24 h after delivery. A similar result was found in a study in which women received guidance on postural adjustments for NB care and activities of daily living [27], in which lower pain scores were observed after the first 2 days following the cesarean section.

Notably, physiotherapeutic interventions used in the postpartum period should include early mobilization, teaching appropriate techniques for standing and sitting that limit contractions of the rectus abdominis muscles, and teaching ideal positions for breastfeeding [27], to reduce pain scores and improve functional rehabilitation.

The importance of reducing pain scores during walking after applying the protocol is highlighted. Pain can be a limiting factor, and early walking should be encouraged to avoid the risk of developing deep‐vein thrombosis [21, 22].

In addition to the effectiveness results, higher satisfaction rates among postpartum women with pain management and control with physiotherapy interventions stand out. This result highlights the importance of physiotherapy in helping reduce discomfort through specific, nonpharmacological physiotherapy resources [10]. However, it is recognized that the support and guidance received may have positively influenced the subjective perception of pain, reinforcing the emotional and psychological aspects of the painful experience and underscoring the importance of support.

This study has several limitations, which are listed below.

Of the 40 women included in the sample, eight (20%) underwent a cesarean section after the onset of labor, and the remaining (80%) were elective. Hormonal differences due to labor, fatigue, and exhaustion may influence the perception of postoperative pain, which can be considered a limitation of the study. It is recommended that future studies consider this variable to improve sample homogeneity.

Seven women (17.5%) had comorbidities; however, at the time of delivery, all were controlled. However, it is recognized that their occurrence may influence pain perception, postoperative recovery, and postoperative functional capacity. Although it was not considered in the analysis, it is necessary to assess the potential impacts on the postpartum woman’s functional limitations.

The same aspect can be observed in relation to a previous cesarean section. In the study, 8 (20%) had undergone surgery previously; half (*n* = 04) had a history of 1 cesarean section, and the other half (*n* = 04) had a history of 2 cesarean sections. The body may respond differently after multiple surgical procedures, which was not considered in the analyses and constitutes a limitation. However, consideration of previous surgical procedures is recommended in future studies on postcesarean pain.

Although the aspects mentioned represent limitations, it is noteworthy that the study focused on evaluating the impact of a physiotherapy intervention protocol on the prevention and reduction of postcesarean pain scores. However, these limitations represent issues to be explored in future studies.

As a limitation of the scientific literature, the small number of studies detailing physiotherapy actions in the immediate postpartum period stands out, especially with a focus on stimulating functional movement to prevent painful postures after cesarean section. This scarcity compromises the broad comparability of data and reinforces the need for further scientific research. Despite this, the present study contributes by proposing a structured and validated protocol with direct practical application for the target audience.

The study’s limitations include a small sample size and a short follow‐up period, as poorly adapted postural changes can persist throughout the postpartum period and impact long‐term functional recovery. Therefore, the results presented should be interpreted with caution. Future research should involve a larger population to confirm these findings. Thus, longitudinal studies with larger samples and a multicenter design are recommended to increase the external validity of the findings and enable more robust generalizations.

The study results showed a significant reduction in pain during walking but without major impacts on other movements, such as those related to breastfeeding. This may be due to the small sample size and short follow‐up period. These findings reinforce the need for further studies with larger sample sizes and longer follow‐up periods.

Another limitation is that this is an open‐label study, meaning it was not blinded. It is recognized that the absence of blinding can introduce detection and performance bias, especially in self‐reported outcomes. This limitation was taken into account in the interpretation of the results, and caution is recommended when generalizing the conclusions. Future studies may consider designs with independent assessors or additional blinding strategies to strengthen internal validity.

Although participant satisfaction with the intervention was measured, the possibility of social desirability bias is acknowledged. This is due to the open‐label study design and to participants being directly questioned by the researchers. It is suggested that, in future studies, satisfaction measurement should be carried out using anonymized, double‐blind instruments.

However, although they represent a limitation, these issues also represent an opportunity for future investigations, considering the benefits that pain reduction can provide to the functionality of postpartum women.

The study made it possible to investigate pain during movement in functional activities, map locations, and evaluate scores. Thus, the results may contribute to the implementation of changes in the physiotherapeutic approaches used in the care of women in the immediate postpartum period, to promote the reduction and control of postcesarean pain and increase maternal well‐being. It is believed that systematic guidance on the execution of appropriate movements that favor the reduction of pain in the most common activities in this period may facilitate the reestablishment of functionality, improving the quality of life of postpartum women.

Although the protocol’s application time (40–50 min) may, at first glance, represent an operational obstacle in high‐turnover maternity wards, its evaluation should consider cost‐effectiveness and the overall impact on care. Nonpharmacological interventions in pain management can reduce the need for pharmacological analgesia, minimize associated adverse effects, and improve relevant subjective outcomes, such as maternal satisfaction and a positive perception of the birth experience.

From an economic and organizational point of view, the investment of professional time can be offset by the reduction of additional interventions (pharmacological or invasive), the optimization of the use of therapeutic resources, the potential decrease in complications associated with inadequate pain management, and the improvement of care quality indicators focused on the patient experience.

To promote feasibility in the hospital setting, strategies such as the following are suggested: integration of the protocol into key moments of labor and structuring care flows that incorporate the intervention without overlapping tasks.

However, it is recognized that implementation can be challenging in settings with limited human resources, which may impact its scalability. Thus, it is recommended that future studies include formal cost‐effectiveness analyses (such as cost per unit reduction on the pain scale or cost per gain in maternal satisfaction) and pragmatic designs that evaluate the intervention in real‐world clinical practice settings.

## 5. Conclusion

The physiotherapy intervention protocol effectively reduced pain scores during walking, with effects on coughing and sitting on the toilet, and increased satisfaction with pain management.

Satisfaction with the intervention reinforces the importance of physiotherapy in postcesarean rehabilitation. This study advances by proposing an evidence‐based, validated physiotherapy intervention protocol for this target audience.

Finally, it is recommended that longitudinal studies be conducted to investigate the effectiveness of physiotherapy interventions with an emphasis on stimulating movement in the immediate postpartum period after cesarean section over time, thus allowing a more robust evaluation of the effects of the intervention and the possibility of generalizing the results.

## Author Contributions

Cristiane Rose Rossi Mazzoni and Mariana Torreglosa Ruiz conceived the study. Cristiane Rose Rossi Mazzoni and Mariana Torreglosa Ruiz oversaw the trial management. Cristiane Rose Rossi Mazzoni and Mariana Torreglosa Ruiz performed the statistical analysis. All authors drafted and contributed to the revision.

## Funding

The authors did not receive any financial support for this research.

## Disclosure

All authors approved the final manuscript.

## Ethics Statement

The study obtained ethical approval from the Ethics Committee, under opinion no. 6,843,708 on May 23, 2024. The study was guided by the Guidelines and Regulatory Norms for Research involving human beings, contained in National Resolution 466/2012/CNS/MS, as well as the ethical principles contained in the Declaration of Helsinki. The authors adhered to the CONSORT guideline [13].

## Consent

Informed consent was obtained from all individuals included in this study or their legal guardians.

## Conflicts of Interest

The authors declare no conflicts of interest.

## Supporting Information

Additional supporting information can be found online in the Supporting Information section.

## Supporting information


**Supporting Information** Protocol “Physiotherapeutic interventions for pain relief in women in the immediate postpartum period following cesarean section.”

## Data Availability

The data that support the findings of this study are available from the corresponding author upon reasonable request.
