# Supplementary material for: Physiotherapy Protocol for Pain in the Immediate Postcesarean Postpartum: Randomized Clinical Trial
Source: Obstet Gynecol Int. 2026 Jun 15;2026:5179249. doi: 10.1155/ogi/5179249 (PMC13269826; doi:10.1155/ogi/5179249)
Supplement: Supplementary file 1 — Supporting Information Protocol “Physiotherapeutic interventions for pain relief in women in the immediate postpartum period following cesarean section.” [file OGI-2026-5179249-s001.docx]

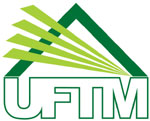


| **Protocol:** Physiotherapeutic interventions for pain relief in women in the immediate postpartum period following cesarean section  **Project:** “Stimulating delivery in the immediate postpartum period following cesarean section to prevent pain resulting from antalgic postures: a randomized clinical trial.”  **Prepared by the research team:**  **PhD. Mariana Torreglosa Ruiz**  **Ms. Cristiane Rose Rossi Mazzoni**  **Collection Center:**  - Federal University of Triângulo Mineiro – University Hospital of the Federal University of Triângulo Mineiro  * This protocol to be tested is based on physiotherapy intervention protocols in the postoperative period of cesarean section in which the proposed activities led to a reduction in pain scores.  It is estimated that the application of this protocol will take approximately 40-50 minutes and should be started after the collection of sociodemographic, clinical, and obstetric data and after the signing of the Informed Consent Form, or the Informed Consent Form when applicable. |
| --- |
| **Responsible parties**: PhD Mariana Torreglosa Ruiz and members of the project team. |
| **Target Audience:** Postpartum women who underwent cesarean section and were assisted in the institution's postpartum wards. |
| **Objectives** |
| To perform physiotherapy interventions to relieve pain during movement in women in the immediate postpartum period following a cesarean section and to evaluate their effectiveness. |
| **Description** |
| **Location:** Rooming-In Wards. |
| **Team required:** researcher trained for intervention. |
| **Inclusion criteria:** All postpartum women who underwent cesarean section, are over 15 years old, hemodynamically stable, conscious, oriented, in good clinical condition according to their medical records, whose procedure was performed more than eight hours prior, after having their urinary catheter removed, and who ate after surgery will be included in the study. |
| **Equipment needed:** a folded sheet or a pillow or cushion if available, a simple chair and/or a chair with arms, if available. |
| **Materials:** 1 small bottle or container with 70% alcohol and/or sink, water and soap for hand hygiene for the researcher; 1 clipboard; pen; Informed Consent Form/Informed Consent Form. |
| **Prerequisites for the protocol** |
| Daily, the researcher will contact the responsible nurse, check the list of hospitalized postpartum women, and, based on the census, identify eligible postpartum women (those who underwent cesarean section).  At the time of contact 0 (allocation), postpartum women over 16 years of age must be hemodynamically stable, conscious and oriented, in good clinical condition, as documented in their medical records, and hospitalized in the Rooming-In Unit. The surgical procedure must have been performed more than eight hours prior, the urinary catheter must have been removed, and the woman must have eaten after surgery.  After identifying eligible participants, the researcher will go to the ward where they are hospitalized. Postpartum women will be informed of the study's objective, and the possibility of participating in the control/intervention groups will be emphasized. If they agree to participate, they will sign the Informed Consent Form.  If the participant is 15-18 years old, she must give her consent, and the Informed Assent Form must be signed by her legal guardian after the necessary explanations.  After signing, the researcher will give the participant one copy of the form and keep the second copy.  Once participation is consented to, the researcher will contact the randomization center defined for the study (which will have the randomization list of participants) via WhatsApp® and indicate the participant's number, receiving the participant's allocation (control/intervention) from the center.  This protocol was not designed to be applied to: women who experience complications/incidents during or after childbirth; who have a clinical contraindication or medical diagnosis of cognitive impairment that prevents participation, as well as those who need to use opioid medications to control pain. These criteria were not designed for exclusionary purposes, but rather to assess the feasibility of the intervention and the follow-up required for the Clinical Trial. However, if the need for postpartum women not included in this protocol is detected, they will receive guidance from the institutional or research team. |
| **Protocol description** |
| Researchers will contact unit nurses to select eligible participants for the study, using data from the daily census of hospitalized patients and medical records. Once the inclusion and exclusion criteria have been verified, they will proceed to the AC wards.  Good morning (or good afternoon), Mrs. XXXX (name of the postpartum woman)! How are you?  Could we speak with you?  My name is Cristiane Rose Rossi Mazzoni. I am a physiotherapist and doctoral student, and I am part of a research team investigating physiotherapy interventions to reduce postpartum pain during movement. I would like to invite you to participate. Could we discuss the study?  **Negative response** – thanks for your attention, and record the refusal.  Affirmative response – that's great! I will read the consent form, which will explain the study. If you have any questions, feel free to interrupt me at any time. At the end of the reading, if you agree to participate, please sign this form in duplicate. I will keep one copy, and the other will remain with you. I inform you that during the research, we will take some notes, should you consent to participate.  **The Informed Consent Form is read aloud.**  The researcher will provide the necessary time for the postpartum woman to discuss her participation with her family/companion.  After obtaining the signature, the form is given to the postpartum woman.  **Precautions before data collection and intervention:**  Ensure the postpartum woman and the newborn are safe (by preventing falls or other incidents).  Ensure there are no physical barriers during contact.  The researcher's cell phone should be on silent mode or turned off at this time.  The researcher who will perform the intervention should be without a watch and always attentive to eye contact with the postpartum woman. For safety reasons, the researcher should not wear jewelry.  The researcher should touch her appropriately, always asking permission, and narrate what is being observed understandably.  Before starting the research itself, the researcher should offer practical support to the postpartum woman – checking whether she has eaten, whether she needs to go to the bathroom, and whether she needs help with the newborn, among other things.  After identifying that she is comfortable, begin the following procedures:  **Procedures:**  The researcher will begin data collection with sociodemographic, clinical, and obstetric questions. These may be supplemented with information from the medical record, if necessary.  Afterwards, she will inquire about functional limitations for each movement type and the pain score during performance. To measure pain, the Numerical Pain Rating Scale (NPRS) will be used. The postpartum woman will be instructed to rate her pain for each activity on a scale of 0 to 10, where 0 indicates no pain, and 10 indicates the worst possible pain.  She will be asked whether she can perform the movement and whether she feels pain while doing so. It is important to clarify that the risk of thrombosis in the lower limbs will be assessed using the Homans Sign and Flag Sign tests before proposing the exercise in item 10 (feet). The following activities will be evaluated:  1. Breastfeeding while lying down  2. Changing from lying down to standing next to the bed  3. Walking  4. Changing and bathing the newborn  5. Breastfeeding/feeding the newborn in a seated position  6. Sitting down/standing up from a chair  7. Sitting down/standing up from the toilet  8. Coughing/sneezing  9. Bathing  10. Active foot movement  If the person reports pain, they will be asked to indicate the point of pain on the image to pinpoint its location.  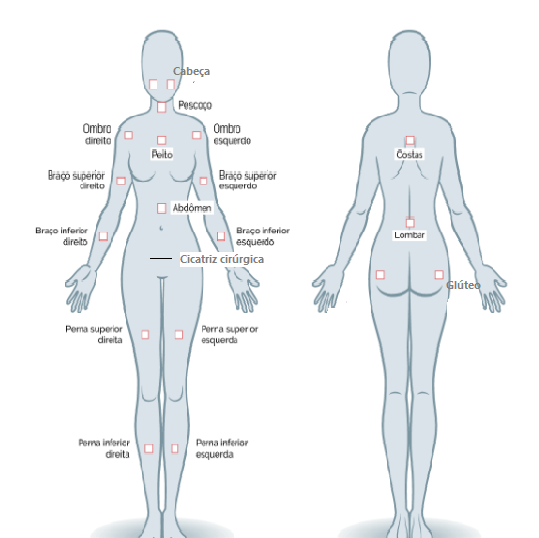  * Figure in Portuguese as per original image.  You will also be asked to describe the sensation of pain, according to the McGill Pain Questionnaire.  **McGill Pain Questionnarie**  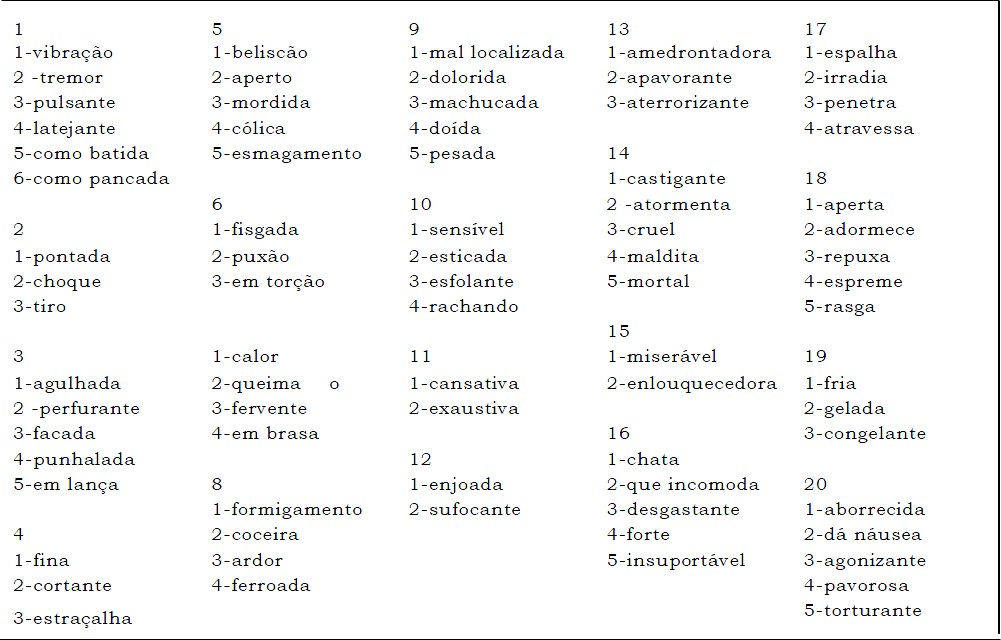Some words that I am going to read describe your current pain. Tell me which words best describe your pain. Choose only one word from each group, the one that best describes your pain (questionnaire adapted for the Portuguese language by Pimenta and Teixeira, 1996.  * Figure in Portuguese as per original image. NÚMERO DE DESCRITORES ÍNDICE DE DORSENSORIAL...................... SENSORIAL.....................   AFETIVO........................... AFETIVO.........................  AVALIATIVO................... AVALIATIVO.................  MISCELÂNIA................... MISCELÂNEA................  **TOTAL........................... TOTAL..........................**  * Figure in Portuguese as per original image.  Subgroups 1 to 10 represent sensory responses to painful experiences (traction, heat, torsion, among others); the descriptors for subgroups 11 to 15 are affective responses (fear, punishment, neurovegetative responses); subgroup 16 is evaluative (evaluation of the overall experience), and subgroups 17 to 20 are miscellaneous.  After collecting this data, the intervention will be carried out according to the following protocol:  **Breastfeeding while lying down:** the postpartum woman will be instructed to adopt the supine position, with or without elevating the head of the bed, respecting her comfort. If the head of the bed is elevated, a pillow or similar object can be placed to support the spine. The newborn should initially be held in one arm, with support from a pillow or similar object under the newborn, on the ipsilateral side to the breast that will be offered. The other arm remains free to assist when necessary. Alternatively, the lateral decubitus position can be adopted with the head supported by a pillow or similar object, or with one arm supporting the head. The other arm can be used to hold the newborn. The head of the bed should be slightly inclined or straight, and the legs can be in semi-flexion, flexion, or extension.  **Changing from a lying to a standing position beside the bed:** A postpartum woman, in a supine position with the bed at an angle, should turn onto her side (the chosen side position can be the one preferred by the postpartum woman) and raise her trunk, supporting herself on the upper limb on the same side as the one supporting her on the bed. At the same time, she should move her lower limbs off the bed before assuming a sitting position. Breathing should be calm, and exhalation will be required during the effort to raise the trunk. In the sitting position, the trunk should be straight, the feet flat on the floor, and the shoulders level, without protrusion. Inhale deeply, drawing air in through the nose and exhaling through the mouth 3 times. Then, place your hands on the bed laterally to your body, directing the force to your legs, and exhale as you stand up. Repeat the inhalation and exhalation 3 times. If the postpartum woman has difficulty performing these movements with the bed not inclined, she can use the head of the bed elevated until she can sit up, then move by rotating her torso and legs to place them off the bed. All movements should be performed during exhalation.  **Walking:** while walking, keep your torso straight, arms along your body, and shoulders relaxed and symmetrical; inhale through your nose and exhale through your mouth at a calm pace; walk for approximately 5-8 minutes.  **Changing and bathing the newborn:** to guide the appropriate height for these activities, the crib in the rooming-in area will be used: patient standing, upright posture with slight flexion of the spine if necessary, the changing and bathing area should be at elbow level, and the patient should not have to bend over to perform the activity;  **Breastfeeding/feeding the newborn in a seated position:** the patient will be instructed to position herself in a chair or similar, with her spine straight and supported by the backrest or, if unavailable, against the wall, and, if available, to use pillows to help support her back. Her feet should be flat on the floor, with her knees at approximately 90°, shoulders level and relaxed, and her cervical spine also relaxed. The newborn can be supported on a pillow or similar item on the mother's lap, where she will cradle him with her arm. The mother will be instructed not to keep looking at the newborn all the time, so that her cervical spine does not remain flexed, avoiding pain. If preferred by the mother, she can adopt the inverted position for the newborn, holding him sideways under the flexed arm with his head supported by the mother's open hand, positioning the newborn's face towards the breast. The baby's back will rest on the mother's forearm.  **Sitting/Standing from a chair:** Begin the activity by using the chair's arms, if available, and the strength of your lower limbs to stand up. Exhale as you sit/stand so that the force is not applied to the abdominal region (the scar). If necessary, support the surgical scar with one hand (palm) and the other hand on the chair to maintain balance while sitting/standing.  **Sitting/Standing from the toilet: To sit:** Concentrate the force on your lower limbs and slowly bend your torso, lowering yourself while exhaling until you reach the toilet. If necessary, use one hand to support yourself on the toilet while lowering yourself onto it. **To stand:** Support yourself firmly on your lower limbs and, if necessary, use one hand (palm) to support the surgical scar. If the strength is insufficient, use one hand to support yourself on the toilet.  **Coughing/sneezing:** Use your hands, palms flat and one on top of the other, to support the scar. A folded towel or pillow/cushion can also be used for support before coughing or sneezing.  **Bathing:** Keep your torso straight, inhale through your nose and exhale through your mouth, support the scar with one hand while using the other to clean your hands. To clean your lower limbs, use a support (chair or similar, if necessary) and raise the limb closest to your hands, avoiding bending your torso.  **Active foot movement:** Patient in supine position, lower limbs above heart level with the bed elevated in the lower limb region, perform alternating active dorsiflexion and plantarflexion movements: 10 repetitions, 2x/day.  After applying the interventions, satisfaction with them will be evaluated using a Likert scale, asking:  How did you feel about the guidance on performing the activities?  ( ) not satisfied ( ) somewhat satisfied ( ) satisfied ( ) very satisfied  The researcher will not take notes during the intervention.  If the baby wants to breastfeed during the intervention, the intervention should be postponed until after breastfeeding.  After the intervention, the researcher will thank the participants, say goodbye, and emphasize that they will return the following day (24 hours after the intervention). If any changes were made to the ward layout, they should be rearranged.  24 hours after the initial contact, the researcher will ask again whether she can perform the movements and record the pain scores for each movement described. If she experiences pain, she will ask her to indicate its location and characteristics.  At the end of data collection, the researcher will hand over a folder containing the instructions for the patient to take home, thank her, and say goodbye. Afterwards, she will check the medical record for analgesic consumption during the period and painful complaints recorded in the medical record. |

**Intervention plan and expected actions**

| **Intervention** | **Expected actions*:** |
| --- | --- |
| The researcher inquires about the pain experienced during movement. If present, the score, location, and characteristics are requested. | - The postpartum woman reports that she has no pain complaints and that she performs the movement without difficulty OR  - The postpartum woman reports that she has pain complaints and that she performs the movement with difficulty. |
| Description of the Intervention | -If the postpartum woman reports no pain and performs the movements without difficulty:  Instructions:  1) Diaphragmatic breathing: 10 repetitions, 2x a day;  After 24 hours, she will be asked again about pain during the movements. If pain is reported, an assessment of the location, pain score, and characteristics will be performed, followed by instructions for the following activities:  1. Breastfeeding lying down  2. Changing from lying down to standing next to the bed  3. Ambulating  4. Changing and bathing the newborn  5. Breastfeeding/feeding the newborn in a seated position  6. Sitting/standing from a chair  7. Sitting/standing from the toilet  8. Coughing/sneezing  9. Taking a shower  10. Active foot movement  Handing out the folder  -If the postpartum woman reports pain:  1) Guidance for performing the following activities:  1. Breastfeeding lying down  2. Changing from lying down to standing beside the bed  3. Walking  4. Changing and bathing the newborn  5. Breastfeeding/feeding the newborn in a seated position  6. Sitting/standing from a chair  7. Sitting/standing from the toilet  8. Coughing/sneezing  9. Showering  10. Active foot movement  2) Diaphragmatic breathing: 10 repetitions, 2x a day  After 24 hours, the patient will be reassessed regarding pain during movements, pain scores for each movement, the location, and the characteristics of the pain.  *Leaflet provided |

**Unexpected situations**

| **Unexpected situations** | **Solutions** |
| --- | --- |
| **Operacional*** | |
| Complications with the postpartum woman during collection/intervention (orthostatic hypotension, malaise) | Stop collecting/intervening, stabilize the postpartum woman, and call a professional from the sector for evaluation. |
| Complications with the newborn (crying, choking, or others) and the postpartum woman being unaccompanied. | Ensure the newborn is safe.  Provide practical assistance.  Call a team member if necessary.  Ensure the postpartum woman is accompanied before starting the collection/intervention. |
| Incidents with the newborn (crying, choking, or others) | Stop collection/intervention, stabilize the newborn, and call a professional from the sector for evaluation if necessary. |
| Communication difficulties (due to low level of education or difficulty understanding terminology) | - Establish a bond with the postpartum woman - Pay attention to signs that the postpartum woman is not understanding or is somehow uncomfortable - Try to make her as comfortable as possible - Simplify the way the guidance is given according to her understanding (comprehension of the words) - Check the companion's understanding and ask for their help. |
| Giving up mid-data collection/intervention | Establish a connection with the postpartum woman, and if, even after explanations, she does not wish to participate, do not insist. |
| The nursing or medical team needs to perform a procedure on the postpartum woman or newborn. | Confirm with the team whether the postpartum woman or newborn will be undergoing any procedures at the time the sample collection/intervention will take place. |
| Visit from other professionals (social worker, nutritionist, physiotherapist) | Confirm with the team whether the postpartum woman will be attending any multidisciplinary appointments at the time the sample collection/intervention will take place. |
| Visiting hours | Confirm visiting hours and conduct data collection/intervention before or after visiting hours. |
| Emergencies involving the postpartum woman, newborn, or the nursing department. | In emergencies, cease collection/intervention, notify the team, assist only if requested, and leave the ward clear. |

**References used to develop the protocol:**

AIN, Q.U.; WAQAR, F., BASHIR, A. Ease in pain and functional activities following caesarean delivery by postnatal exercises (pilot study). **J Islamic Int Med Coll**, Paquistão, v.13, n. 1, p. 5-32, 2018. Available from: https://journals.riphah.edu.pk/index.php/jiimc/article/view/1024. Acesso em: 2 mar. 2025.

ARTAL, R. Guidelines of the American College of Obstetricians and Gynecologists for exercise during pregnancy and the postpartum period. **BrJ Sports Med,** London, UK, v. 37, n.1 p.6–12, 2003. Available from: https://bjsm.bmj.com/content/bjsports/37/1/6.full.pdf Acesso em: 4 fev. 2024.

BURTI, J. S.; CRUZ, J. de P. da S.; SILVA, A. C. da; MOREIRA, I. de L. Assistência ao puerpério imediato: o papel da fisioterapia. **Revista da Faculdade de Ciências Médicas de Sorocaba**, Sorocaba, SP, v. 4, n. 18, p. 193-8, nov. 2016. Available from: https://revistas.pucsp.br/index.php/RFCMS/article/view/25440. Acesso em: 20 mar. 2025. [Portuguese]

INSTITUTO da Criança e do Adolescente. **Posicionamento da mãe/bebê**. Available from: https://icr.usp.br/posicionamento_mae_bebe/. Acesso em: 3 mar. 2025. [Portuguese]

KARAKAYA İ.; YUKSEL, I.; AKBAYRAK, T.; DEMIRTURK, F.; KARAKAYA, M. G.; OZYUNCU, O. et al. Effects of physiotherapy on pain and functional activities after cesarean delivery. **Arch Gynecol Obstet**, Munique, DE, v. 285, n. 3, p. 621–7, 2012. Available from: https://doi.org/10.1007/s00404-011-2037-0. Acesso em: 2 mar. 2025.

KAUR H.; KAUR S.; SIKKA P. A quasi-experimental study to assess the effectiveness of early ambulation in post-operative recovery among post-caesarean mothers admitted in selected areas of Nehru Hospital, PGIMER, Chandigarh. **Nursing Midwifery Res J**, Índia, v. 7, n. 1, p. 55-59, 2015. Available from: https://doi.org/10.33698/nrf0180. Acesso em 2 mar. 2025.

PEREIRA, T. R. C.; MONTESANO, F. T.; FERREIRA, P. D.; MINOZZI, A. S.; BELEZA, A. C. S. Existe associação entre os desconfortos no puerpério imediato e a via de parto? Um estudo observacional. **Abcs Health Sciences**, Santo André, SP, v. 42, n. 2, p.80-84, maio/jun. 2017. Available from: <http://novo.more.ufsc.br>. Acesso em: 3 jun. 2025. [Portuguese]

RETT, M. T.; BERNARDES, N. de O.; SANTOS, A. M. dos, OLIVEIRA, M. R. de, ANDRADE, S. C. de. Atendimento de puérperas pela fisioterapia em uma maternidade pública humanizada. **Fisioterapia e Pesquisa**, São Paulo, v. 15, n. 4, p. 361-66, 2008. FapUNIFESP (SciELO). Available from: https://www.scielo.br/j/fp/a/bzckfKhjXwHg3T66BkrRMCJ/?format=pdf&lang=pt. Acesso em: 3 jun. 2025. [Portuguese]

SABOIA D.M.; BEZERRA K.C.; VASCONCELOS N.J. A.; BEZERRA L.R.P.S.; ORIÁ M.O.B.; VASCONCELOS C.T.M. The effectiveness of post-partum interventions to prevent urinary incontinence: a systematic review. **Rev Bras Enferm** [Internet]. 2018;71 (Supl. 3):1460-8. [Thematic Issue: Health of woman and child]. Available from: http://dx.doi.org/10.1590/0034-7167-2017-0338. Acesso em 25 jun. 2025.
